# Portugal’s first major forensic case and the genesis of forensic toxicology: 10 years of research to reconstruct the event

**DOI:** 10.1080/20961790.2018.1534538

**Published:** 2018-11-20

**Authors:** Ricardo Jorge Dinis-Oliveira

**Affiliations:** a Department of Sciences, IINFACTS – Institute of Research and Advanced Training in Health Sciences and Technologies, University Institute of Health Sciences (IUCS), CESPU, CRL, Gandra, Portugal;; b UCIBIO-REQUIMTE, Laboratory of Toxicology, Department of Biological Sciences, Faculty of Pharmacy, University of Porto, Porto, Portugal;; c Department of Public Health and Forensic Sciences, and Medical Education, Faculty of Medicine, University of Porto, Porto, Portugal

**Keywords:** Forensic sciences, forensic toxicology, Vicente Urbino de Freitas, António Joaquim Ferreira da Silva, Medical-Surgical School, Mário Guilherme Augusto de Sampaio

## Abstract

The “Crime of Flores Street” is one of the most famous cases of poisoning to divide public opinion in Portugal in the late 19th century, and it also demonstrated the weaknesses of the Portuguese medicolegal system and attested to the importance of toxicological analysis. Vicente Urbino de Freitas was a prominent doctor, graduating from the Faculty of Medicine of the University of Coimbra in 1875. He later became Professor of Physiology at the Porto Medical-Surgical School and author of a number of books on leprosy. In 1877, he married Maria das Dores Basto Sampaio Freitas, and this was followed by the death of a number of her close relatives in suspicious circumstances, notably her brother José António Sampaio Junior and nephew Mário Guilherme Augusto de Sampaio. This review aims to retell the story of Portugal’s first significant medicolegal case as well as the accompanying judicial drama that gave birth to Forensic Toxicology in Portugal and prompted the medicolegal organization that exists today. This research was carried out over a 10-year period and represents undeniable historical value given the rarity of the facts compiled. At the heart of this forensic case was the use of toxicological analyses in court for which the Chemist António Joaquim Ferreira da Silva played a key role. This toxicological report revealed high concentrations of morphine, delphinine and narceine in viscera and in Mario’s urine. The Mario’s cause of death was attributed to poisoning by opium alkaloids. Despite the strong judicial evidence, doubts still remains as to whether Vicente Urbino de Freitas was a “monster” or a victim of circumstances and a hapless martyr.

## Introduction

**Figure 1.**
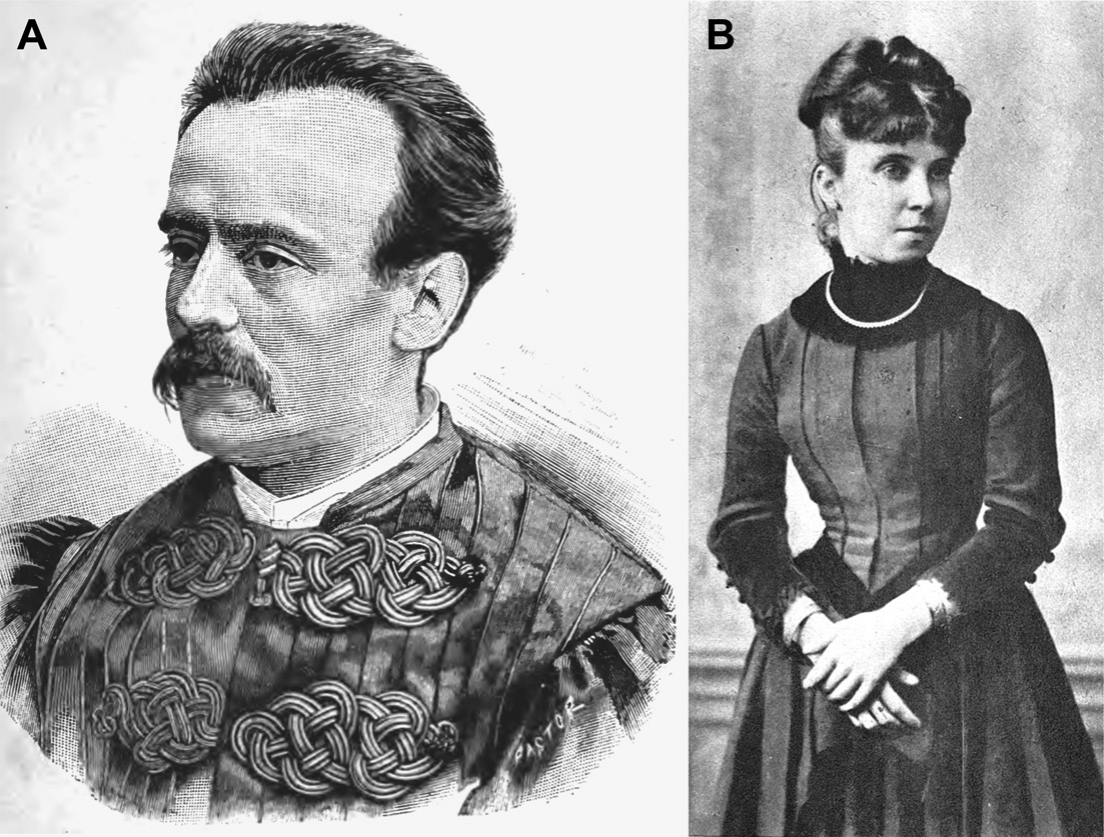
Portraits of Vicente Urbino de Freitas (A) and his wife Maria das Dores Basto Sampaio Freitas (B).

**Figure 2.**
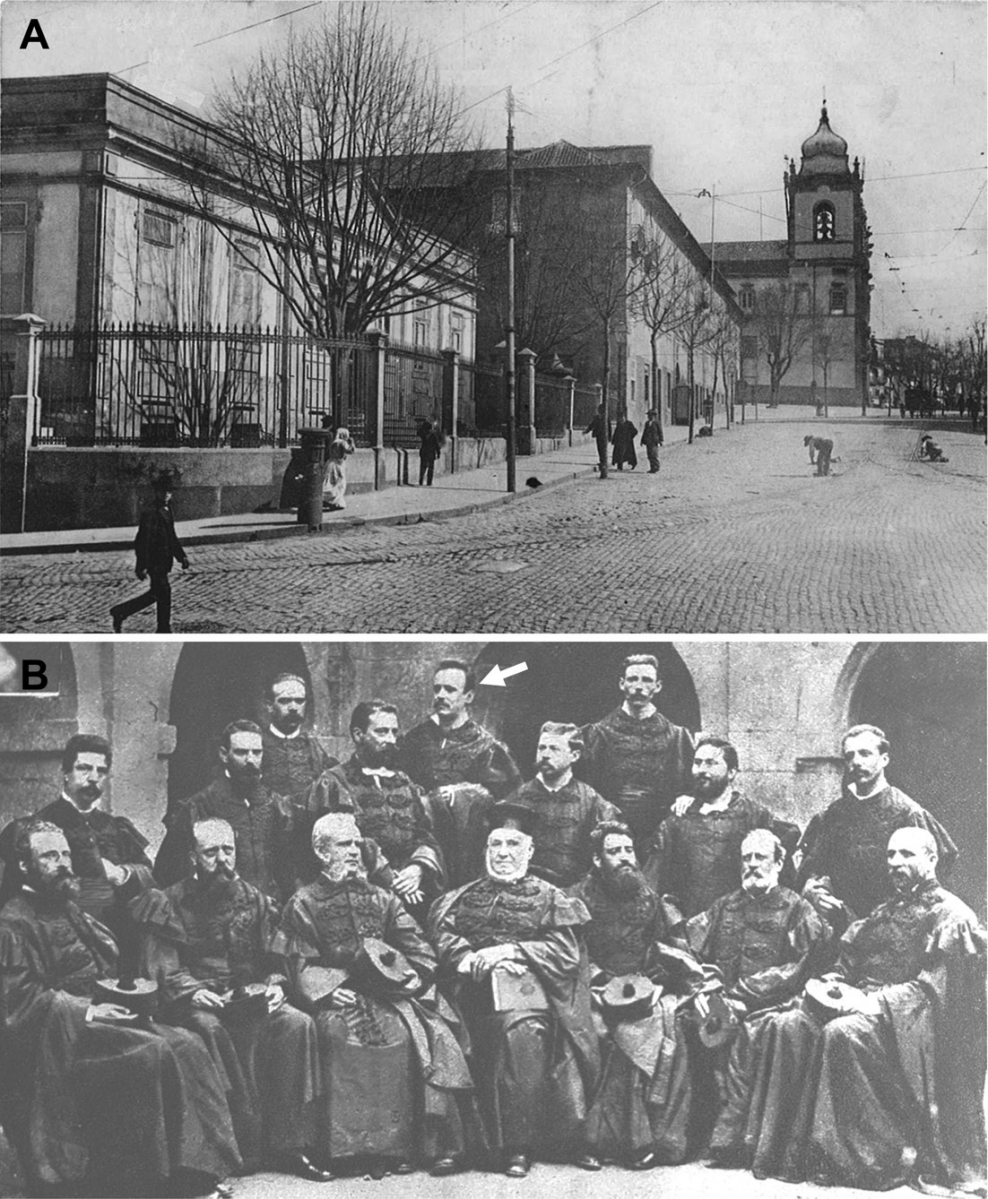
(A) Porto Medical-Surgical School (1836–1911); (B) Group of professors at the Medical-Surgical School of Porto, 1881. Arrow shows Vicente Urbino de Freitas.

**Figure 3.**
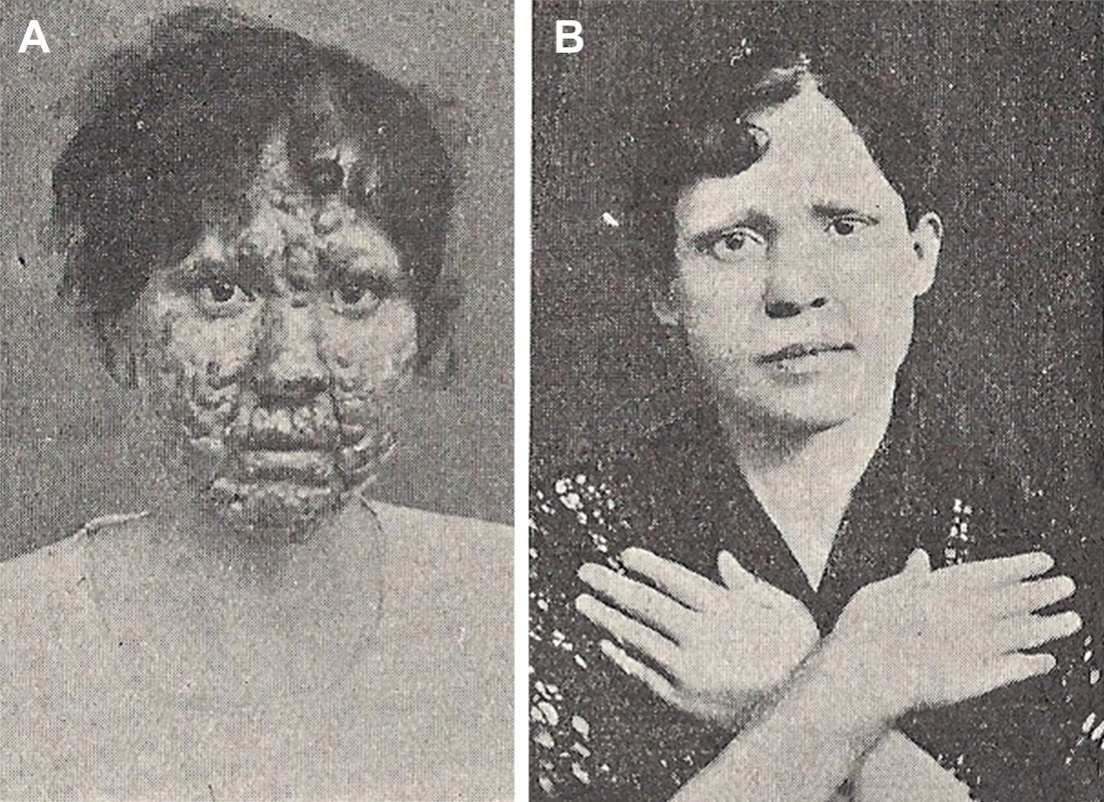
Patient of Vicente Urbino de Freitas with leprosy (A) and recovery after 2 months of treatment (B).

The genesis of Forensic Toxicology in Portugal is closely related to the restructuring of the Portuguese medicolegal services. The first major medicolegal case in Portugal both fascinated and stunned Portuguese society in the late 19th century. The controversy provoked by the medical research relating to the trial of Vicente Urbino de Freitas served to rekindle the debate about the reform of medicolegal services in Portugal, which had been long demanded and promised. Vicente Urbino de Freitas (Figure 1A) was born in Flores Street, Porto on August 31, 1849. In 1877, he married Maria das Dores Basto Sampaio Freitas (Figure 1B), daughter of José António Sampaio, a wealthy linen merchant also from Porto. Vicente Urbino de Freitas graduated by the University of Coimbra and 2 years later he obtained a lecturing position at the Medical-Surgical School of Porto under a decree dated September 6, 1877 (Figure 2A and B). He was distinguished by his notable studies on dermatology, namely concerning the treatment of leprosy (Figure 3A and B), a subject to which he devoted part of his clinical practice. In a graphical abstract, Vicente Urbino de Freitas is shown wearing the uniform of the Medical-Surgical School of Porto. However, he would later see his name recorded in the history of Porto and Portugal for less notable reasons. In April 1890 he left the Medical-Surgical School of Porto, where he had gained scientific recognition and social prestige, and was taken to Porto Cadeia da Relação (i.e. prison) on suspicion of having poisoned his nephew Mário Guilherme Augusto de Sampaio [1]. The judicial process lasted for more than 3 years, during which rumours circulated that he had committed other murders, including that of his newborn daughter and other relatives. He was found guilty of the crime in 1893 and spent the next 20 years away from Porto: he served his prison sentence in Lisbon, and this was followed by deportation to Angola and then exile. During this period he also experienced a number of personal tragedies, including the suicide of his son Urbino Emílio Basto de Sampaio Freitas, who was unable to bear the stigma his father’s crimes committed against his family members. After the libertarian period of the Republic of Angola, the restrictions against Vicente Urbino de Freitas were lifted and in September 1913, he decided to return to Porto in the German packet-boat König Friedrich August with the aim of proving his innocence. Upon his return, he immediately gave an in-depth interview to “Jornal da Tarde” [The afternoon Journal]. The story was published in two parts on September 30 and October 1, 1913: the articles presented the portrait of a domineering man who refused to let interviewers unravel the mysteries of his life.

At the heart of this case was the use of toxicological analyses in court. The chemist António Joaquim Ferreira da Silva played a key role in this process and further demonstrated a great commitment to improve the quality of the teaching Toxicology in Portugal. Because of its innovative aspects, the toxicological investigation in the case of Vicente Urbino de Freitas is considered by some authors to represent the true beginning of Portuguese Forensic Toxicology. This article presents the first major Portuguese medicolegal case while also explaining the great judicial drama that promoted Forensic Toxicology in Portugal and initiated the medicolegal organization that exists today.

## Material and methods

The bibliographical research inherent to this reconstruction began in the middle of 2007. It entailed 10 years of research in libraries studying historical works and the discovery of the transcript of the 1893 criminal hearing and those newspapers that published various reports about the event. The research was not limited to literature in Portuguese (from Portugal and Brazil) but was also included English and French documents given the worldwide coverage of this forensic case [1–9].

## The sequence of historical events

**Figure 4.**
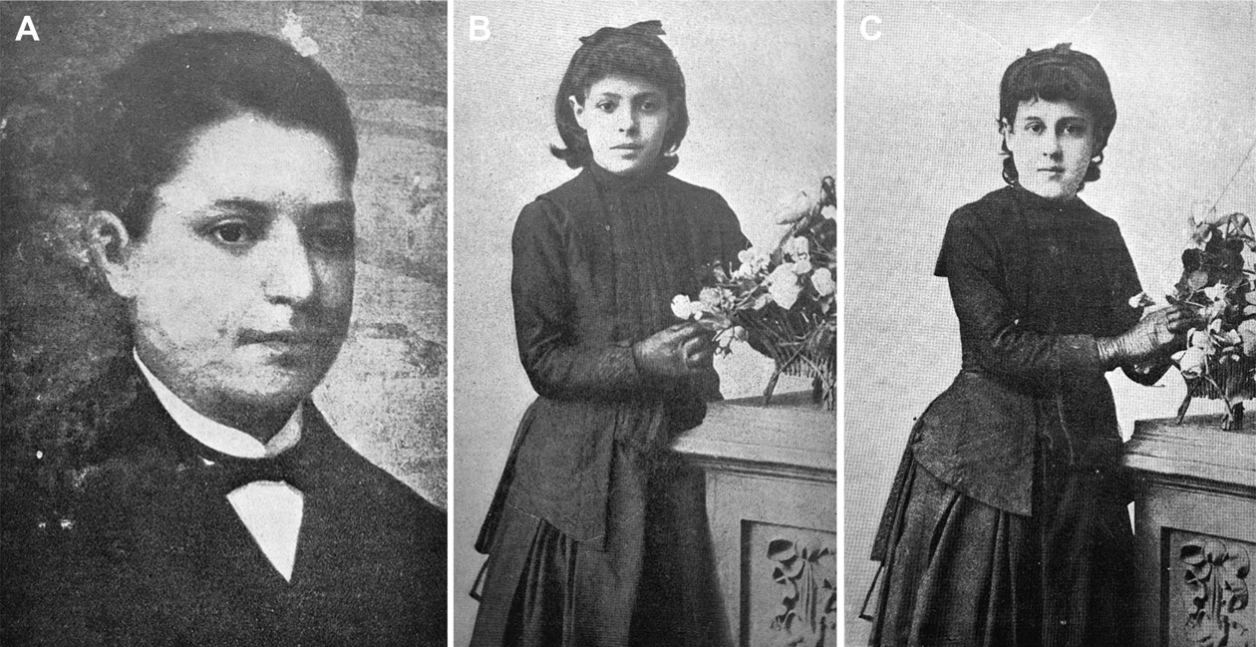
Portraits of Mário Guilherme Augusto de Sampaio (A), Maria Augusta Sampaio (B) and Berta Fernanda Sampaio (C).

**Figure 5.**
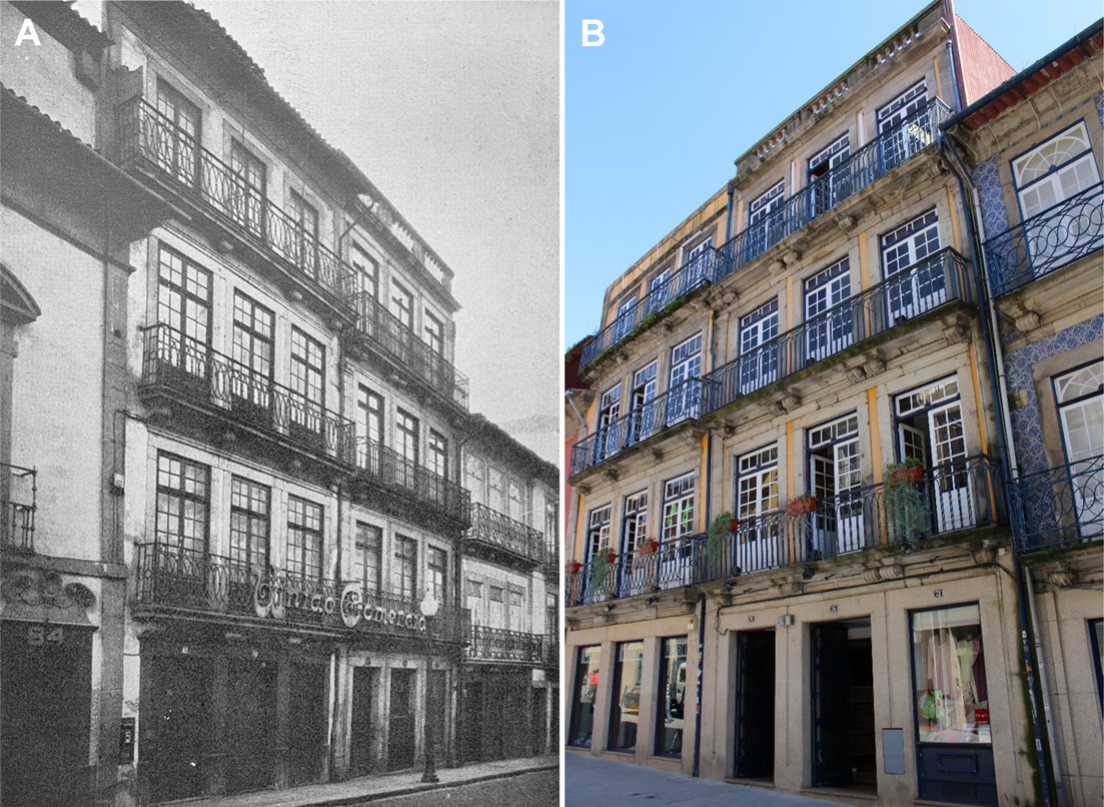
House of Vicente Urbino de Freitas’s in-laws located at Flores Street, Porto, where Mário Guilherme Augusto de Sampaio was poisoned. Photographed at the time of the case (A) and today (B).

José António Sampaio, a rich and prestigious linen merchant, and his wife Maria Carolina Bastos Sampaio lived in Flores Street, Porto. They had three children: Guilherme Sampaio, José António de Sampaio Junior and Maria das Dores Basto Sampaio Freitas. Vicente Urbino de Freitas’s brother-in-law Guilherme died shortly after his marriage to Maria das Dores Basto Sampaio Freitas, leaving behind two children, Mário Guilherme Augusto de Sampaio (Figure 4A) and Maria Augusta Sampaio (Figure 4B). The wife of José António de Sampaio Junior also died young, leaving a daughter, Berta Fernanda Sampaio (Figure 4C) who went to live with her cousins Mário and Maria Augusta at their grandparents’ house (Figure 5A and B).

Vicente Urbino de Freitas’s brother-in-law José António de Sampaio Junior, a bohemian, came to Porto with Miss Karter Lothie (an English woman from a nightclub in Chiado, Lisbon), and settled into the city’s oldest hotel (i.e. Grand Hotel de Paris situated in Fábrica Street). He had the misfortune to fall sick and made the fatal error of seeking help from his prestigious brother-in-law. José died on January 2, 1890, showing typical signs and symptoms of poisoning, and with Vicente Urbino de Freitas recording that he was in terrible agony with “blood in his vomit”.

**Figure 6.**
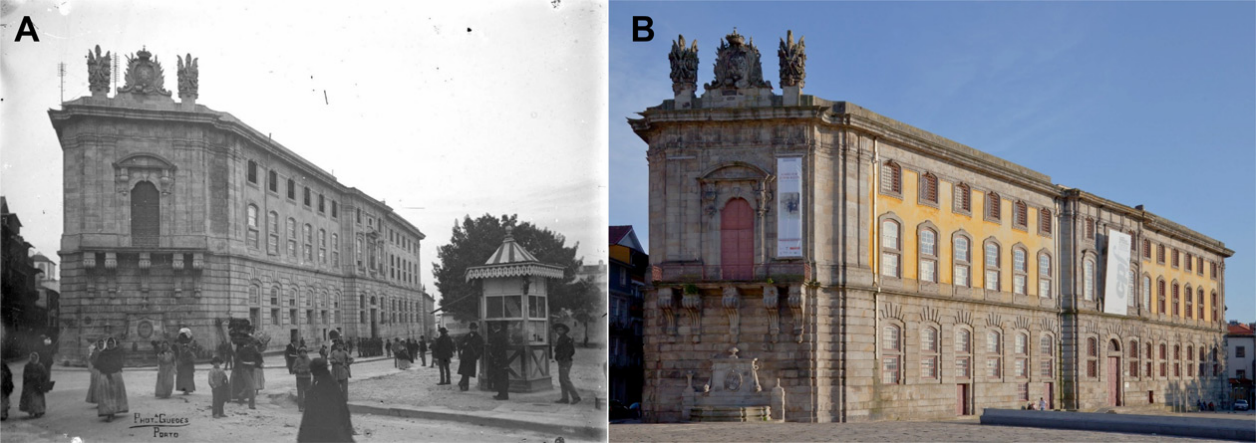
Porto Cadeia da Relação (i.e. prison; actual Portuguese Centre of Photography) where Vicente Urbino de Freitas was held in cell 13. Photographed at the time of the case (A) and today (B).

**Figure 7.**
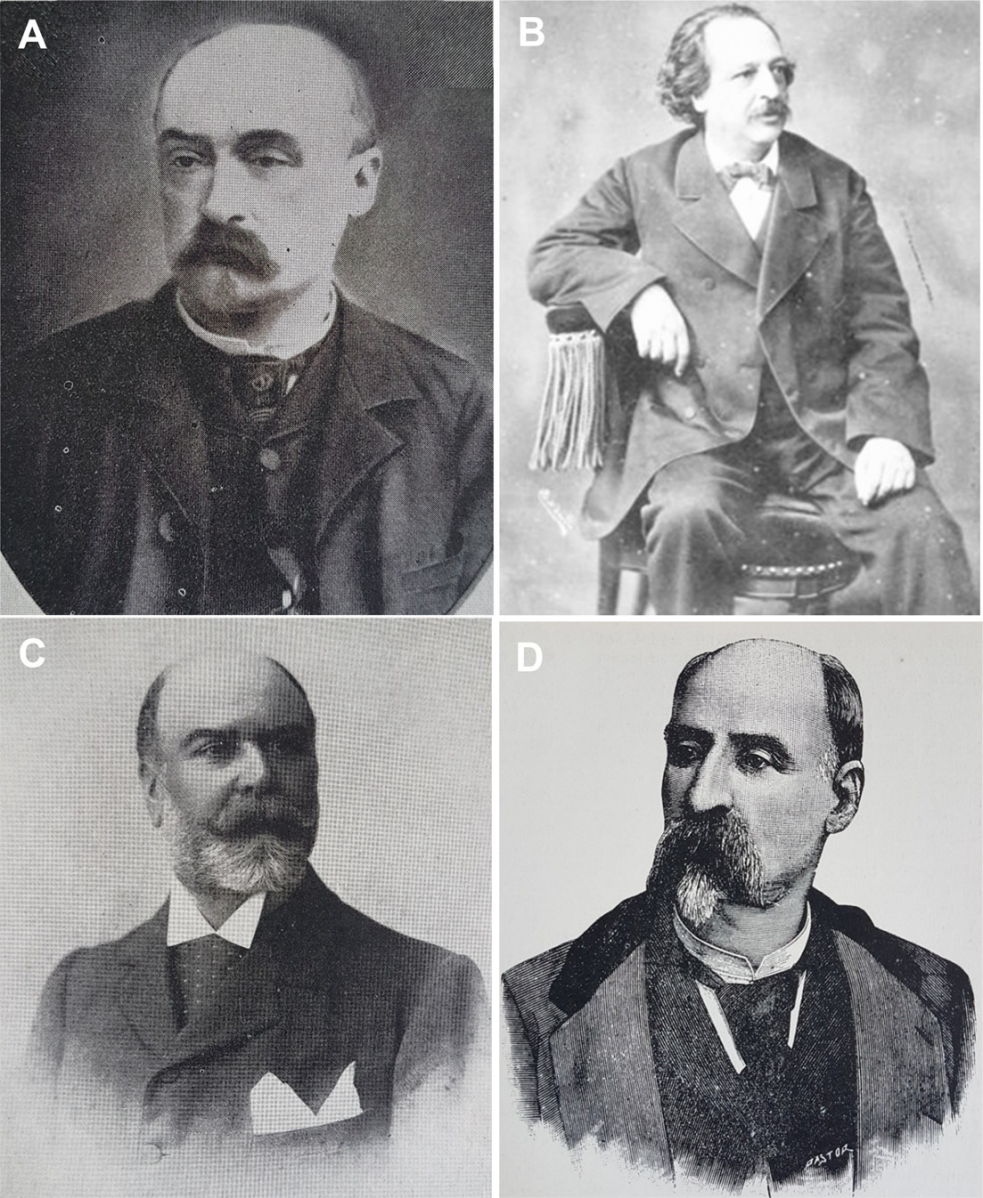
Portraits of the defence attorneys João Carlos Freire Temudo Rangel (A) and Alexandre Braga (B), the public prosecutor Miguel Maria de Guimarães Pestana da Silva (C) and the judge Ernesto Kopke de Fonseca e Gouveia (D).

On Saturday March 29, 1890, the eve of Palm Sunday, a mysterious package addressed to Berta Fernanda Sampaio was delivered to Flores Street coming from Lucio Martins (an unknown sender) it contained almonds with liquor and precisely three coconut and chocolate cakes, one for each of the children living at the house. The children’s grandmother Maria Carolina Bastos Sampaio was reluctant for the children to eat the cakes but relented and on Monday March 31, they ate the cakes and soon began to feel unwell. On Tuesday April 1, Vicente Urbino de Freitas was called and he prescribed them lemon balm clysters, “urging them to make a peristaltic retention as long as possible”. The oldest child, 12-year-old Mário Guilherme Augusto de Sampaio, died on April 2, 1890 experiencing spasms and convulsions, similarly to his uncle José Sampaio. Suspicions of poisoning, first from the cakes and then with the lemon balm, fell on Vicente Urbino de Freitas, as he had reason to find ways to eliminate any competition to becoming the heir to his father-in-law’s fortune. According to the testimonies of several other physicians, they had never seen lemon balm herbal remedies used to treat signs and symptoms such as those evidenced in the children. This case became known as the infamous “Crime of Flores Street”, with the circumstances of the crime and the coldness and cruelty of the acts causing much excitement and indignation. On April 16, 1890, following a number of inconsistencies in his testimony, Vicente Urbino de Freitas was arrested by the General Police Commissioner in Porto Cadeia da Relação (Figure 6A and B). He was held for almost 3.5 years in cell 13 at the back of the building. The lawsuit was filed on April 23, 1890, with Vicente Urbino de Freitas charged with murder by poisoning, committed against Mário Guilherme Augusto de Sampaio. João Carlos Freire Temudo Rangel (Figure 7A) and Alexandre Braga (Figure 7B) were Vicente’s defence attorneys, the public prosecutor was Miguel Maria de Guimarães Pestana da Silva (Figure 7C) and Ernesto Kopke de Fonseca e Gouveia (Figure 7D) was the judge. Some of the other cells at Porto Cadeia da Relação also contained illustrious prisoners. For example, cell 12 was occupied by the writer Camilo Castelo Branco; Vicente Urbino de Freitas later became his friend and doctor, and his cell has been preserved and still exists today. Some documents relating to the criminal proceedings of Vicente Urbino de Freitas can be found in the Judiciary Museum housed in Porto’s Justice Palace, currently home to the Court of Appeal. The original court documents, approximately 2 800 sheets and 5 600 handwritten pages, are held in the Porto District Archive.

## Toxicological experts and analyses

The main forensic issues surrounded the toxicological analyses of the corpses and the suspected poisoned foods. A group of four experts was assembled: (i) Agostinho António do Souto, a professor at the Medical-Surgical School of Porto; (ii) Joaquim Pinto d’Azevedo, a surgeon and anatomy expert at the same school; (iii) Manoel Rodrigues da Silva Pinto, a professor of Legal Medicine and Toxicology at the Medical-Surgical School of Porto; and (iv) António Joaquim Ferreira da Silva (1853–1923, Figure 8), a professor and the Director of the Polytechnic Academy of Porto (now the Rectory of the University of Porto; Figure 9A), which existed between 1837 and 1911, and also the Director of the Municipal Chemistry Laboratory of Porto (Figure 9B), founded in 1884. Based on the construction of the current Aliados Avenue, the Municipal Chemistry Laboratory of Porto was closed and demolished in 1916 [3]. Of the above four experts, António Joaquim Ferreira da Silva, who graduated from the Faculty of Philosophy of the University of Coimbra in 1876, deserves special mention. Although he was invited to remain at the University of Coimbra as a lecturer, he declined, preferring instead to apply for the Polytechnic Academy of Porto, where he began working in 1877. He founded the Portuguese Society of Chemistry and published numerous articles in the field of Analytical Chemistry. However, he gained significant international recognition for his work in Toxicology, especially in Forensic Toxicology/Chemistry. He is most well-known for his contribution to the discovery of the characteristic reactions of cocaine and physostigmine and the refinement of the reagent used in the detection of morphine and codeine, which later became known as the Lafon and Ferreira da Silva reagent [4]. He was member of the Medicolegal Council of Porto and in 1902 published a work titled “The Teaching of Toxicology and Pharmacy Reform” [7].

**Figure 8.**
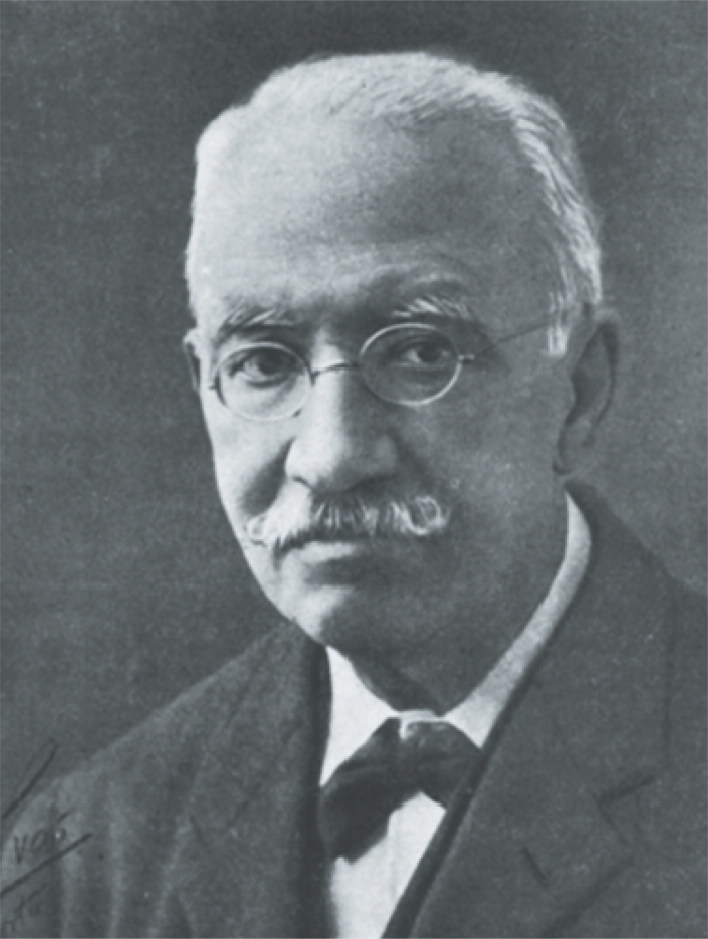
António Joaquim Ferreira da Silva.

**Figure 9.**
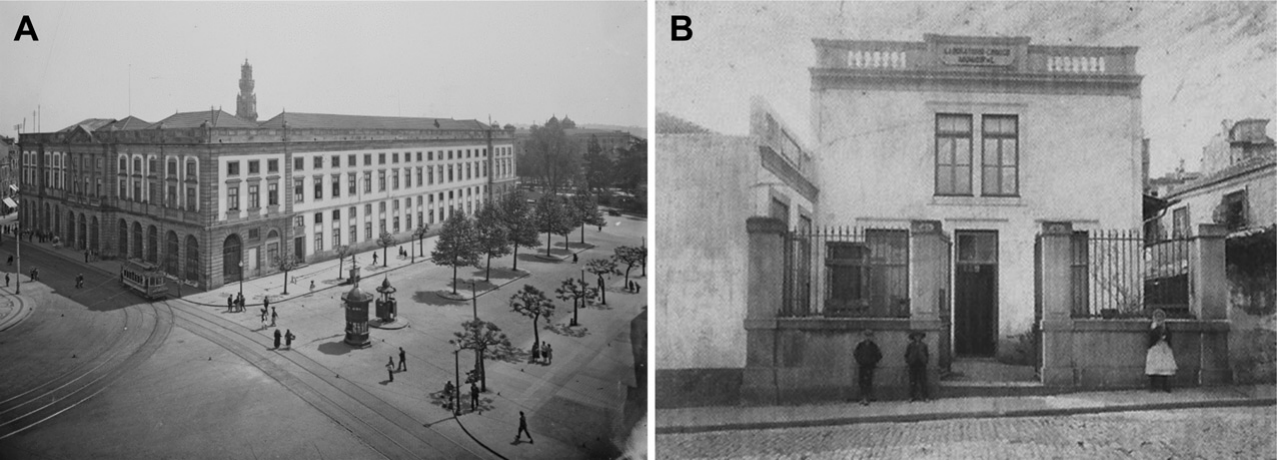
Polytechnic Academy of Porto (A) and Municipal Chemistry Laboratory of Porto (B).

Faced with the growing rumours about suspected poisoning, physicians Dr. Rodrigo de Sousa Moreno and Dr. Alcino Ferreira da Cunha exhumed the corpse of José António de Sampaio Junior from the Agramonte Cemetery on April 10, 1890 and performed an autopsy. Having been buried for 3 months and 8 days, they found him “in an advanced state of decomposition”, making it impossible to observe any signs of injury relating to a cause of death [8,9]. Hence, the forensic experts collected the almost liquefied remains of the encephalic mass, stomach, intestines, lungs and heart for toxicological analysis at the Municipal Chemistry Laboratory of Porto. The first autopsy of Mário Guilherme Augusto de Sampaio was performed on April 4 by Dr. Franchini and Dr. Adelino Costa, and the corpse was then exhumed on April 17 for a second autopsy.

**Figure 10.**
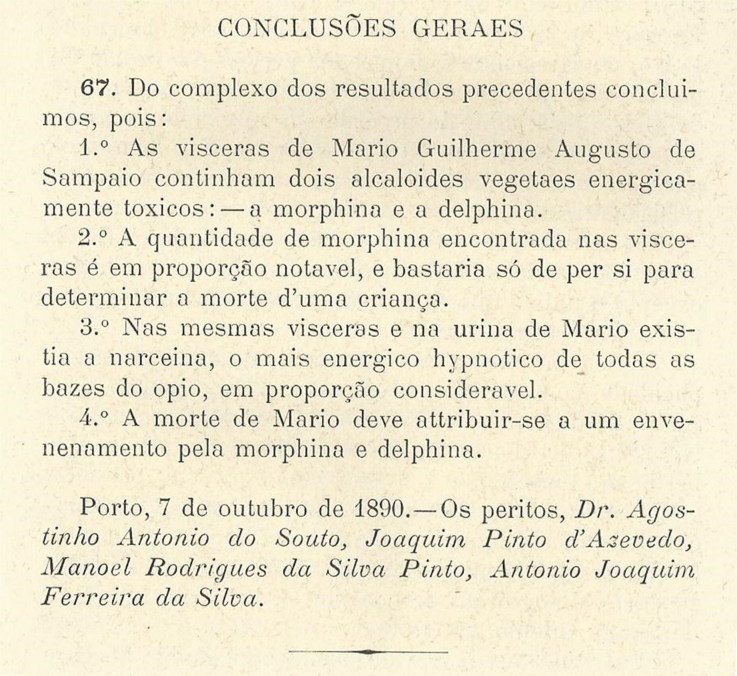
Results of the first major Portuguese toxicological investigation.

According to the forensic report (Figure 10) written by the four experts and presented on October 7, 1890, alkaloids were not detected in the viscera of José António Sampaio Junior, a situation attributed to the advanced state of decomposition. The report also contained information about the viscera of Mário Guilherme Augusto de Sampaio: (i) two toxic plant alkaloids were detected: morphine and delphinine; (ii) morphine was reported to be in high concentrations, enough to cause the death of a child; (iii) narceine (i.e. also an opium alkaloid) was found also “in considerable proportions” in the viscera and in Mario’s urine; and (iv) Mario’s death was attributed to morphine and delphinine poisoning [8,9]. Tests on the almonds did not reveal the presence of any toxic substance. Thus, Portugal’s first significant toxicological forensic results were produced [8,9].

## The intervention of other national and foreign forensic experts

**Figure 11.**
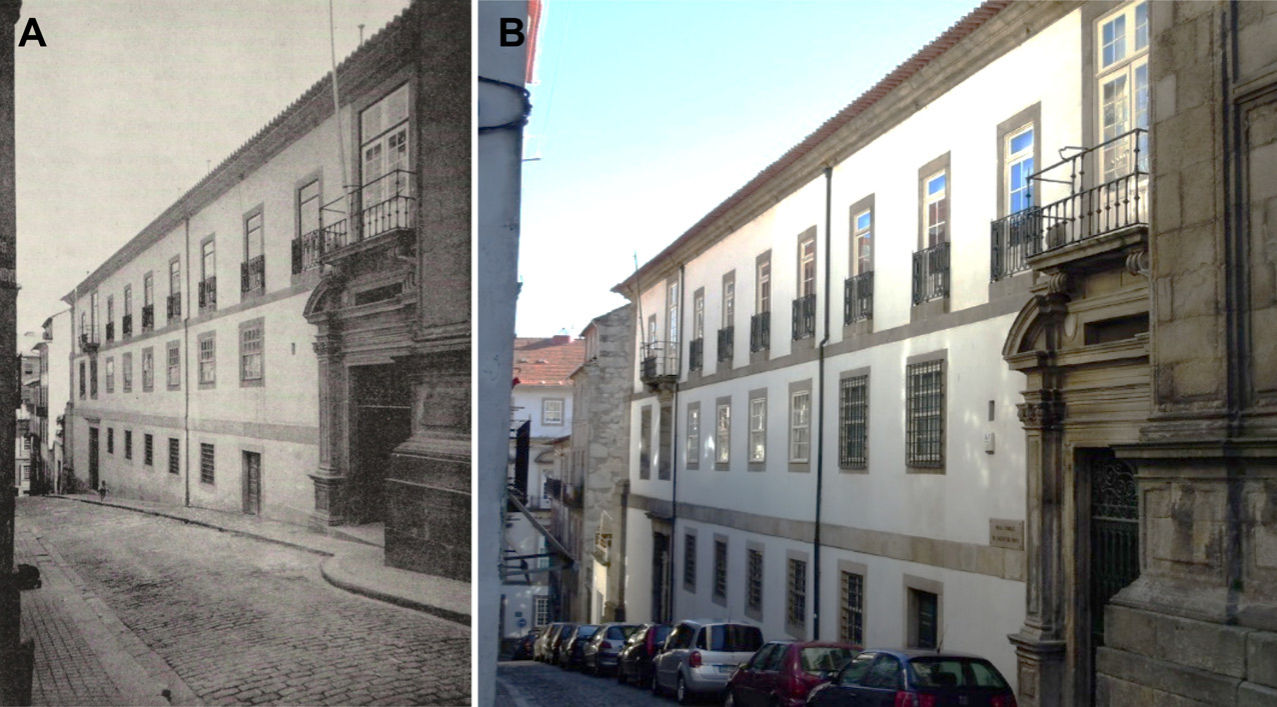
Criminal Court of São João Novo do Porto. Photographed at the time of the case (A) and today (B).

Vicente Urbino de Freitas’s brother, João António de Freitas Fortuna (businessman and the editor of Camilo Castelo Branco’s books among others), fought to prove Vicente Urbino de Freitas’s innocence in court. However, despite significant efforts, Vicente Urbino de Freitas was found guilty on December 1, 1893 in the Criminal Court of São João Novo do Porto (Figure 11A and B). Based on Article 353 of the Penal Code, the court handed down the following sentence for the murder of his nephew Mário Guilherme Augusto de Sampaio: 8 years’ imprisonment (starting May 28, 1894 at the Lisbon Penitentiary) and 20 years deportation to Luanda, Angola (at the beginning of 1901), or, alternatively, 28 years deportation with a period of imprisonment of 8–10 years at the place of deportation. João António de Freitas Fortuna opened an account at the Bank of England, offering a reward for anyone who could prove Vicente Urbino de Freitas’s innocence. Other people also believed his innocence, and Ana Plácido, Camilo Castelo Branco’s wife, offered him asylum at her home in São Miguel de Seide (Vila Nova de Famalicão), whereby Vicente Urbino de Freitas could escape to Spain to prepare a fair legal defence.

**Figure 12.**
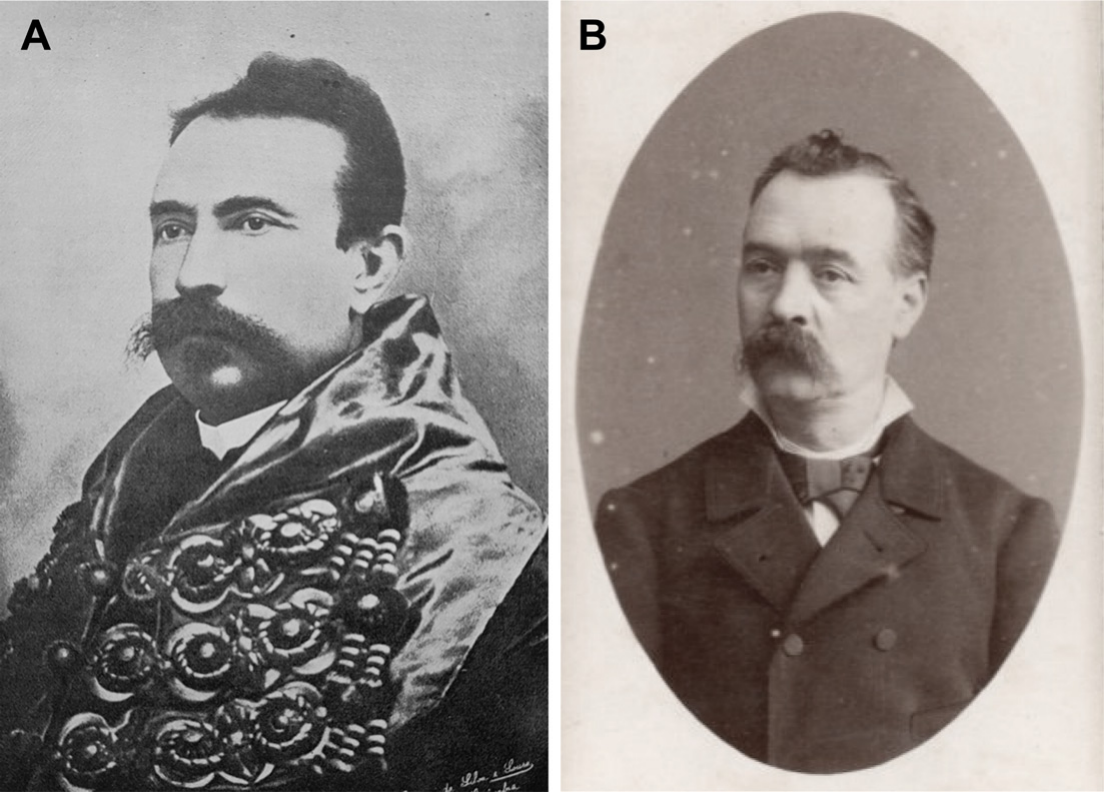
Portraits of Augusto António Rocha Peixoto (A) and Joaquim dos Santos e Silva (B).

The controversy increased when Vicente Urbino de Freitas’s defence lawyers, following the appeal to the Porto Court of Appeal, recruited a physician and professor from the Faculty of Medicine of the University of Coimbra, Augusto António Rocha Peixoto (1849–1901; Figure 12A). He was a former faculty colleague of Vicente Urbino de Freitas, and he who agreed to help out on two conditions: (i) he would need to consult eminent foreign toxicologists namely Dr. Carl Bischoff and Dr. Ludwig Brieger (professors at the University of Berlin), Dr. Heinrich Beckurts (a professor at Brunswick Polytechnic School) and Dr. Hugo Mastbaum (an analytical chemist at the Faculty of Sciences of Berlin, who at that time resided in Portugal as an analytical chemist and Director of the Chemistry Laboratory of the 7th Agronomic Region of Lisbon) and (ii) also work with Joaquim dos Santos e Silva (1841–1906; Figure 12B), a highly respected pharmacist at the Chemistry Laboratory of the University of Coimbra. The latter was responsible for conducting toxicological and chemistry–legal analyses ordered by the Courts of Coimbra between 1878 and 1899. In a series of articles published in *Coimbra Médica*, the two professors from the University of Coimbra criticized and discredited the medicolegal report and its proponents. This then started a war between the experts from Porto and those from Coimbra, a war that would be won by the former, at least from a legal point of view. Indeed, the decision of the Porto Court of Appeal on February 3, 1894 increased the sentence confirmed by the Supreme Court of Justice, sentencing Vicente to 9 years in prison, followed by 20 years deportation accompanied by 2 years prison, or, alternatively, 30 years deportation, including 10 years’ imprisonment at the place of deportation. Among other criticisms, experts from Coimbra concluded that,

the chemical reactions in no way demonstrate the existence of alkaloids, which experts say they have found. The reagents used in the chemical reactions were also impure. Regarding urine, the analyses often result in flagrant confusions; and concerning viscera in view of the admissibility of the presence of products of cadaveric putrefaction, we cannot accept the conclusions drawn by the experts… [8,9].

Of particular interest is the correspondence exchanged with the well-known professor Johann Georg Noel Dragendorff, whose method to detect low-concentration alkaloids is still used today. Dragendorff agreed with his German colleagues.

The courts in all three instances accepted the validity of the conclusions of the expert investigations and coupled with the abundant testimonial evidence, although naturally circumstantial given the nature of the crime and the modus operandi, the defendant was convicted. Indeed, in light of the scientific knowledge at the time and the procedural rules in force, any other decision would not have been possible in this famous case. The judges diligently fulfilled their duty to apply the law, handing down a penalty deemed appropriate for a person recorded in history as a cold, calculating and ambitious serial killer seeking to eliminate all challengers to the future inheritance of his wife. He was described as a “monster” [10].

**Figure 13.**
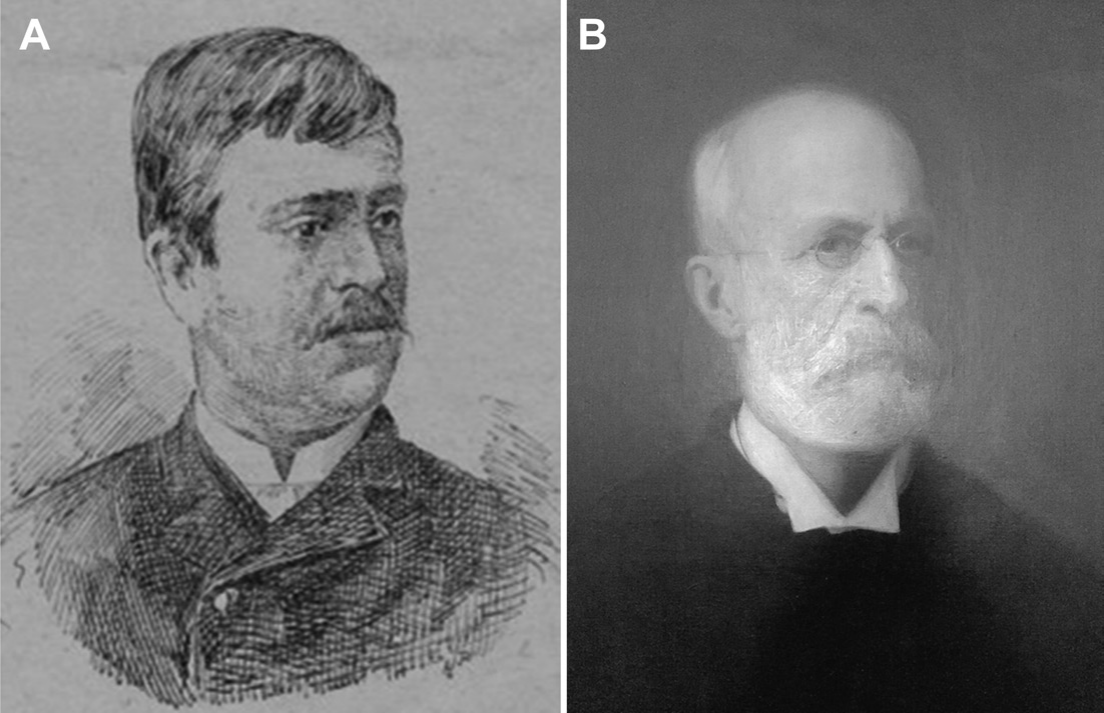
Portraits of José António de Sampaio Junior (A) and José Frutuoso Aires de Gouveia Osório (B).

It came out during the trial that Vicente Urbino de Freitas was also suspected of being involved in the deaths of the banker Roriz, his brother-in-law, José António de Sampaio Junior (Figure 13A) and Dr. José Frutuoso Aires de Gouveia Osório (Figure 13B) but no evidence was produced. The latter was a professor at the Medical-Surgical School of Porto and the first responsively for the Curricular Unit of Public Hygiene and Legal Medicine (established in 1863). He died on August 23, 1887 and the “Gouveia Osório Room” at the Museum of the History of Medicine Maximiano Lemos of the Faculty of Medicine of the University of Porto is named in his honour.

While serving his sentence in Luanda, Vicente Urbino de Freitas received in 1904 a pardon from King Charles on the condition that he not reside in any Portuguese territories. Thus, he voluntarily exiled himself to Rio de Janeiro (Brazil), where he tried to reopen his case and prove his innocence. In Brazil, Vicente Urbino de Freitas twice sought permission to practice medicine, first in Campinas and then in Rio de Janeiro; both applications were rejected. In 1906 in Rio de Janeiro, there was a government clamp down on illegal medical practices. Thus, the Director of Public Health, Oswaldo Gonçalves Cruz (1872–1917) ordered the prosecution of Vicente Urbino de Freitas for illegally practicing medicine and wrote to the local pharmacies prohibiting them from filling his prescriptions. Vicente’s failure to comply with these orders resulted in his expulsion from Brazil. Five days before he was to leave he was arrested because the Supreme Court annulled a writ of *habeas corpus* granted by the federal court, judging the magistrate incompetent to rule on the unconstitutionality of the expulsion law. A group of Portuguese and Brazilians, convinced of Vicente Urbino de Freitas’s innocence, then sent a petition to King Carlos requesting a review of the case, but this was not granted.

**Figure 14.**
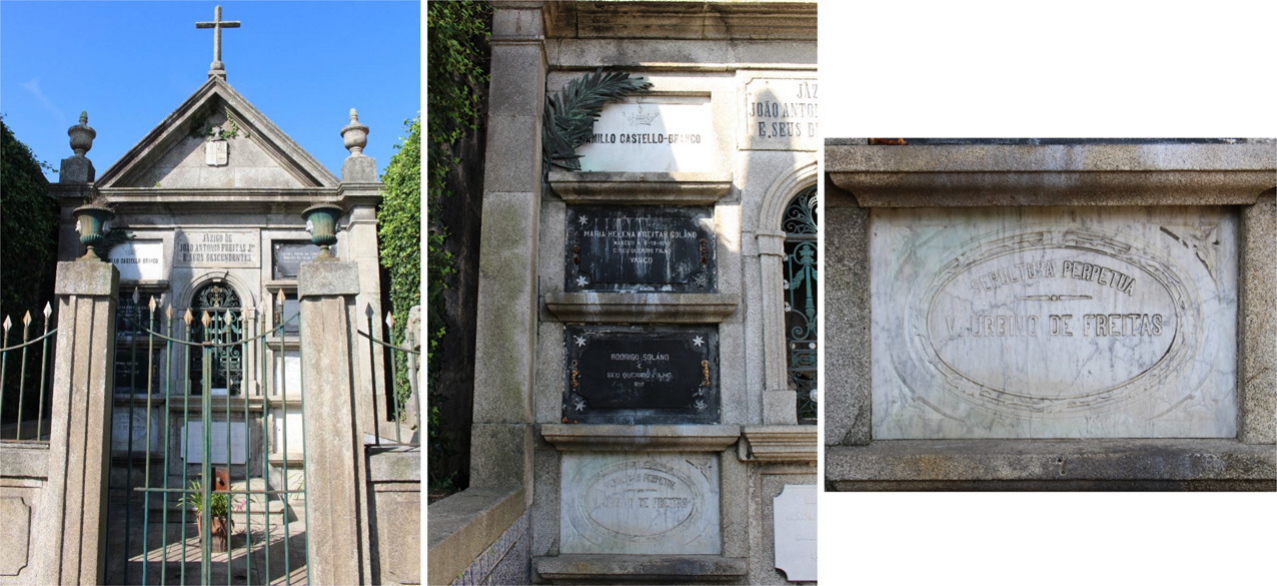
Chapel of the Freitas Fortuna family where Vicente Urbino de Freitas and Camilo Castelo Branco are buried.

Vicente Urbino de Freitas died of pneumonia in Benfica on October 23, 1913, less than a month after returning to Portugal while awaiting a judicial review. He was buried in Lapa cemetery, taking with him any possibility of resolving the mystery surrounding the poisonings (Figure 14). José António de Freitas Fortuna was a close friend of Camilo Castelo Branco, and on July 15, 1889 Camilo wrote to his friend stating that he wished to be buried with Vicente Urbino de Freitas:

I confirm, by this letter, what I proposed to him with reference to my corpse and its deposit in Lapa cemetery. I wish to be buried perpetually in your chapel. My dear Freitas accepted with brotherly tenderness the offer of my corpse, allowing me to be part of your sleeping family.

And there they rest, as does Maria das Dores Basto Sampaio. Thus, Camilo Castelo Branco did not wish to be buried in the National Pantheon in Lisbon.

## Vicente Urbino de Freitas: guilty or innocent?

**Figure 15.**
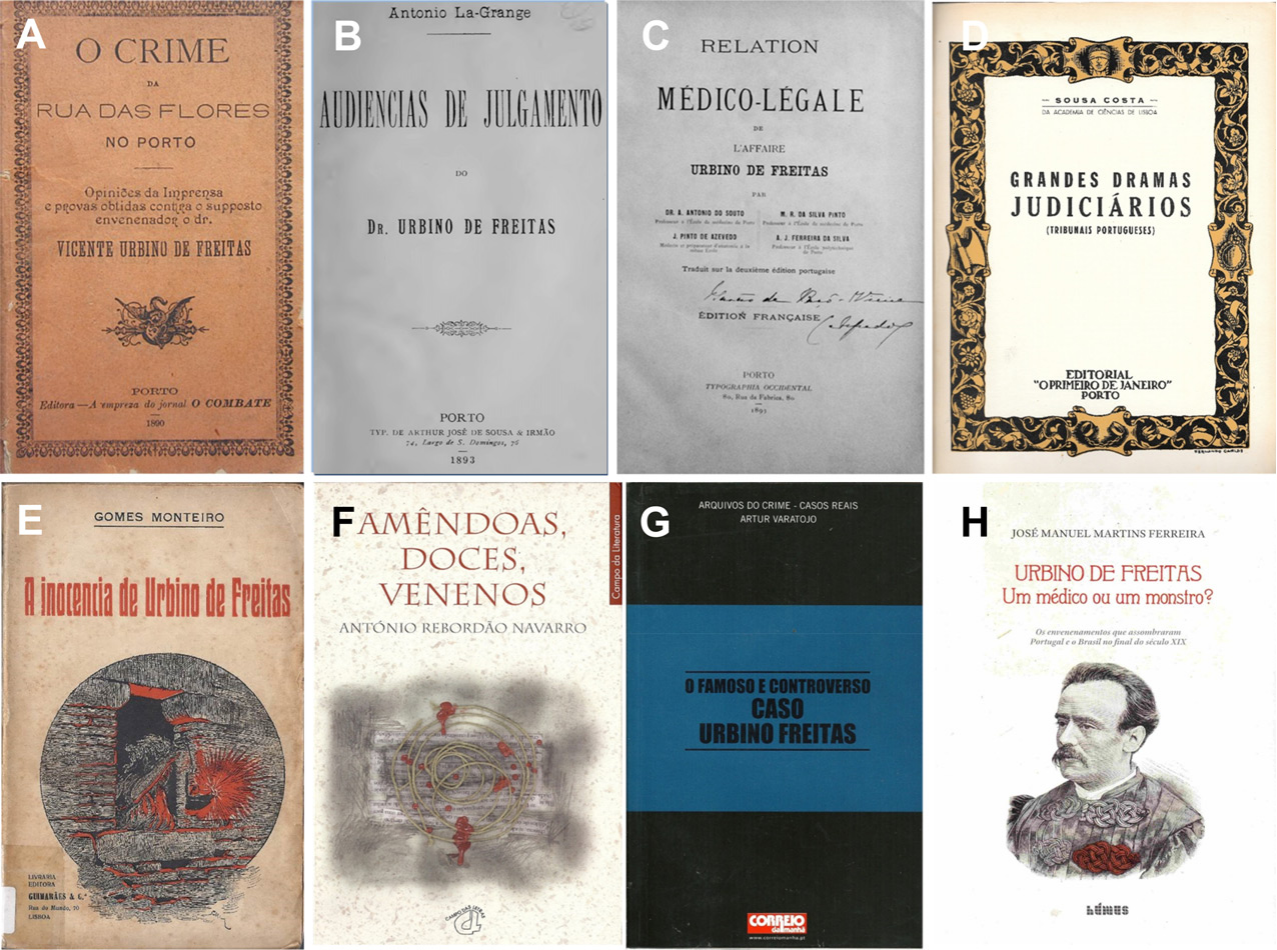
Historical literary works consulted and acquired from various countries. O crime da Rua das Flores no Porto: opiniões de imprensa e provas obtidas contra o suposto envenenador o dr. Vicente Urbino de Freitas [The Crime of Flowers Street in Porto: Press Reviews and Evidence Obtained Against the Supposed Poisoner Dr. Vicente Urbino de Freitas] (A); As audiências de julgamento do Dr. Urbino de Freitas [Court Hearings of Dr. Urbino de Freitas] (B); O caso médico-legal Urbino de Freitas [The Medicolegal Case of Urbino de Freitas] (C); Grandes dramas judiciários (Tribunais Portugueses) [Great Judicial Dramas (Portuguese courts)] (D); A inocência de Urbino de Freitas [The Innocence of Urbino de Freitas] (E); Amêndoas, doces, venenos [Almonds, Sweets, Poisons] (F); O famoso e controverso caso Urbino Freitas [The Famous and Controversial Case of Urbino Freitas] (G); Urbino de Freitas. Médico ou um monstro? [Urbino de Freitas: A Doctor or a Monster?] (H).

Was Vicente Urbino de Freitas really a criminal? According to Gomes Monteiro in his 1933 book “A Inocência de Urbino de Freitas” [The Innocence of Urbino de Freitas] (Figure 15E) [6], one of the main reasons for his conviction was the unusual interest taken by the public prosecutor Miguel Maria de Guimarães Pestana da Silva (Figure 7C), who had previously been an unsuccessful suitor of Maria das Dores Basto Sampaio. Was this detail the decisive factor for the condemnation of the illustrious physician? Or was it a strategy to confuse the prosecution and public opinion? The truth is that Vicente Urbino de Freitas did not flee and doubts still persist, especially from an expert point of view because the unmistakable detection of morphine, narceine and delphinine seems somewhat very difficult in light of the scientific advances of that time. Also strange is the apparent careless behaviour and naivety of the murderer, behaviour that completely contradicts the personality of a man like Vicente Urbino de Freitas. Indeed, he was graduated from the Faculty of Medicine of the University of Coimbra, received honourable academic distinctions and possessed many patients because of his excellent reputation. Moreover, it should be noted that at the first trial the jury responded to the presented facts by returning a majority verdict. Thus, a lack of unanimity shows that some jurors were not convinced of his guilt.

The defendant’s mother-in-law, Maria Carolina Basto Sampaio, made dramatic statements at the hearing and trial, obviously a source of strong emotions. At one point she said in despair,

I swear here before God and men that it was this man who killed my son José and my grandson Mario! It was this man whom I gave budget to go abroad to learn about poisons to kill my family! [11]

It is easy to imagine the impact of such statements on the sensibilities of the jurors, *ad hoc* actors, who had been placed into an unfamiliar environment with no judicial experience.

The jury in criminal proceedings, when required, was formed by selecting 10 citizens by ballot. Thus, the roles of the jury and the judge were very different [11]. The judge, firmly committed to adhering to professional ethics, critically and objectively analyses every document, expert examination and all testimonial evidence as it is produced, reaching the end of the trial with a solidly formed conviction. In contrast, lawyers speak primarily to their clients and the public. The jurors, often with emotion and curiosity, and sometimes confused, follow the events from the chairs as if watching a reality show. In regard to the jurors, the lawyers claim and try to be more convincing than their opponent, as well as being as factious and emotional as possible. At least one advisory judge, Martins Teixeira, intervening in the decision of the Supreme Court of Justice had doubts, and he considered that the nullity of the guilty verdict was due to the failure to request the collaboration of foreign toxicologists, as the defence had intended. Despite the strong judicial evidence, doubts still remains as to whether the “monster” was indeed a victim of circumstances and a hapless martyr [11].

## Conclusions and future perspectives

Motivated by the social standings of the families involved, this forensic case garnered significant national and international attention. On the one hand it involved a prestigious physician, and on the other, the Portuguese forensic expertise that was, for the first time, put to the test. The high level of interest resulted in the publication of a number of articles including “O crime da Rua das Flores no Porto: opiniões de imprensa e provas obtidas contra o suposto envenenador o dr. Vicente Urbino de Freitas” [The Crime of Flowers Street in Porto: Press Reviews and Evidence Obtained Against the Supposed Poisoner Dr. Vicente Urbino de Freitas] (Figure 15A) [12] and the “Audiências de julgamento do Dr. Urbino de Freitas” [Court Hearings of Dr. Urbino de Freitas] (Figure 15B) [5]. Because of the controversy stemming from the toxicological investigation, the full toxicological reports were published in “O caso médico-legal Urbino de Freitas” [The Medicolegal Case of Urbino de Freitas] (Figure 15C), written by the forensic experts of Porto. This book had both a significant national and international impact (*i.e.,* it was also published in French), broadening the visibility of Toxicology as a science [8,9]. Indeed, the experts also sought, as they themselves stated, to enlighten the public and demonstrate the impartiality and professionalism of the performed toxicological investigation. The general public discussed the case on a daily basis and it garnered significant worldwide interest with both *Le Figaro* and *The Times* reporting on the case. In 1944, the case of Vicente Urbino de Freitas was included in “Grandes dramas judiciários (Tribunais Portugueses)” [Great Judicial Dramas (Portuguese courts)] (Figure 15D) [2]. Later in 1998, António Rebordão Navarro published the novel “Amêndoas, doces, venenos” [Almonds, Sweets, Poisons] (Figure 15F) and in 2003 Artur Varatojo wrote “O famoso e controverso caso Urbino Freitas” [The Famous and Controversial Case of Urbino Freitas] (Figure 15G) [13]. A new work on the matter was published in 2018, “Urbino de Freitas. Médico ou um monstro?” [Urbino de Freitas: A Doctor or a Monster?] (Figure 15H), written by engineer José Manuel Martins Ferreira, and attracting enormous public and historical interest [10].

Although not involved in this case, in this final note it is important to make reference to another renowned Portuguese forensic toxicologist, António da Costa Simões (1819–1903). He established Forensic Science at Coimbra University and was responsible for the detection of toxic substances in suspected poisonings. In a series of articles published in *The Institute* in 1855, the physician and professor at the Faculty of Medicine described in detail the methods used in the analysis of samples from real cases, revealing an unusual scientific rigor at the time. In 1860, António da Costa Simões published a toxicology manual that was subsequently used at the School of Medicine in the following decades. His initiatives in Forensic Sciences would be pursued by his classmate and friend José Ferreira de Macedo Pinto (1814–1895), physician and professor at the Legal Medicine, Public Hygiene and Medical Police Department of the Faculty of Medicine of the University of Coimbra. He was responsible for the founding of the Chemistry Office of the Faculty of Medicine, which at that time (1860) already had a vast collection of reagents, instruments and tools for toxicological analysis. In 1860 José Ferreira de Macedo Pinto published a book entitled “Toxicologia judicial e legislativa” [Legal and Legislative Toxicology], with the intention that it be “to be a textbook in the teaching of this science and practical guide for toxicological examinations, to elucidate magistrates, lawyers and jurors” [14].

The limitations of this study are those inherent to the difficulties of bibliographical research and the financial costs associated with the acquisition of centennial works. The information now presented deals with a textual corpus of singular characteristics, never before collated or studied. It represents 10 years of research, searching for information not found in classical databases such as PubMed, but instead in museums and libraries scattered around the world, and in online platforms such as OLX. Thus, I consider this research to be my civic duty toward a scientific area that I embrace, especially when the case concerns the history of my country.

The case of Vicente Urbino de Freitas is a landmark in the genesis and dissemination of Toxicology in Portugal. In 1899, medicolegal services in Portugal were finally addressed under an initiative of the Minister of Justice Jose d’Alpoim. The Carta de Lei [Law Letter] of August 17, 1899 divided the country into three medicolegal service areas (Porto, Coimbra and Lisbon) based at the cities’ morgues (this decision was very controversy at that time). They functioned together with the respective Faculty of Medicine of Coimbra and the Medical-Surgical Schools of Lisbon and Porto. Only autopsies and forensic clinical evaluations were performed. Thus, they served both the interests of justice and education, and this comfortable relationship still remains.

Until the end of his life, Vicente Urbino de Freitas was involved in a legal battle, seeking new evidence to enable him to obtain a favourable judicial decision. However, he never succeeded in obtaining a review of his case. Despite this, he had the never-ending support and unbreakable faith of his extraordinary wife, Maria das Dores Basto Sampaio Freitas, who died aged 97 years on May 18, 1956 at her Benfica home. I conclude with the words uttered near the end of his life by Camilo Castelo Branco in a letter addressed to João António de Freitas Fortuna in solidarity with the situation of his brother: “on leaving this horrendous world, I offer you two words: courage and hope. Human justice should receive from divine justice a ray of light that reaches its abyss. Goodbye, my disgraced friend!”

## Compliance with ethical standards

This article does not contain any studies with human participants or animals performed by any of the authors.
